# Comparison of 1-Ethyl-3-(3-Dimethylaminopropyl) Carbodiimide Based Strategies to Crosslink Antibodies on Amine-Functionalized Platforms for Immunodiagnostic Applications 

**DOI:** 10.3390/diagnostics2030023

**Published:** 2012-08-27

**Authors:** Sandeep Kumar Vashist

**Affiliations:** 1 Centre for Bioanalytical Sciences, National Centre for Sensor Research, Dublin City University, Dublin 9, Ireland; 2 Bristol-Myers Squibb (BMS), Swords Laboratories, Watery Lane, Swords, Co. Dublin, Ireland

**Keywords:** EDC, NHS, sulfoNHS, antibody crosslinking, APTES-functionalized platforms, ELISA, SPR

## Abstract

1-Ethyl-3-(3-dimethylaminopropyl) carbodiimide (EDC) alone, and in combination with N-hydroxysuccinimide (NHS) or sulfoNHS were employed for crosslinking anti-human fetuin A (HFA) antibodies on 3-aminopropyltriethoxysilane (APTES)-functionalized surface plasmon resonance (SPR) gold chip and 96-well microtiter plate. The SPR immunoassay and sandwich enzyme linked immunosorbent immunoassay (ELISA) for HFA clearly demonstrated that EDC crosslinks anti-HFA antibodies to APTES-functionalized bioanalytical platforms more efficiently than EDC/NHS and EDC/sulfoNHS at a normal pH of 7.4. Similar results were obtained by sandwich ELISAs for human Lipocalin-2 and human albumin, and direct ELISA for horseradish peroxidase. The more efficient crosslinking of antibodies by EDC to the APTES-functionalized platforms increased the cost-effectiveness and analytical performance of our immunoassays. This study will be of wide interest to researchers developing immunoassays on APTES-functionalized platforms that are being widely used in biomedical diagnostics, biosensors, lab-on-a-chip and point-of-care-devices. It stresses a critical need of an intensive investigation into the mechanisms of EDC-based amine-carboxyl coupling under various experimental conditions.

## 1. Introduction

The immobilization of antibodies on the bioanalytical platforms is the most critical step in immunodiagnostics as it directly impacts their analytical performance [1]. A wide range of antibody immobilization strategies [2,3,4,5] are available such as physical adsorption, orientated binding by intermediate proteins, covalent binding, biotin-avidin interactions, affinity tags, and site-specific binding. However, the strategies based on the covalent binding of antibodies are the most prominent as they lead to rapid, leach-proof and highly stable antibody binding with high immobilization density. The most widely used covalent binding strategy is the heterobifunctional crosslinking of the amino or carboxyl groups on antibodies to the free carboxyl or amino groups on bioanalytical platforms using EDC along with NHS or sulfoNHS. We have employed a wide range of antibody crosslinking strategies for immunodiagnostic applications. It was observed that the crosslinking of antibodies by their amino groups impacts their antigen detection due to their improper orientation because the amino groups are present at different sites on the antibody including the region near the antigen-binding site. Therefore, in all our immunodiagnostic applications, we crosslink the antibodies by their carboxyl groups, which provides a favorable orientation as the carboxyl groups are located on the fragment crystallizable region of the antibodies away from their antigen binding site. In the present study on APTES-functionalized bioanalytical platforms, various EDC-based antibody crosslinking chemistries are compared, where EDC binds initially to the carboxyl groups on the antibodies followed by the subsequent formation of amide bonds with the amino groups present on the surface. NHS or sulfoNHS is used to stabilize the intermediate in the crosslinking reaction.

While the combination of EDC with NHS and sulfoNHS (EDC/NHS and EDC/sulfoNHS respectively) based biomolecular immobilization strategies have been widely employed for assay development [6,7,8,9,10,11,12,13,14,15,16,17,18,19], EDC by itself has not been used so extensively. To our knowledge, this is the first report that shows the effect of various EDC-based antibody crosslinking strategies on the analytical performance of immunoassays that were performed on APTES-functionalized bioanalytical platforms. Human fetuin A (HFA) immunoassays were performed on anti-HFA antibody-bound APTES-functionalized SPR gold (Au) chip and 96-well microtiter plate (MTP). HFA immunoassay was taken as all the components were commercially-available in the form of a sandwich ELISA kit from R&D Systems, USA. Similar experiments were also performed on two other sandwich ELISAs for human Lipocalin-2 and human albumin, and a direct ELISA for horseradish peroxidase (HRP). The results obtained from all these immunoassays clearly demonstrated that EDC crosslinks antibodies more efficiently on APTES-functionalized bioanalytical platforms than EDC/NHS and EDC/sulfoNHS at the normal pH of 7.4. Therefore, there is a critical need to elucidate the exact mechanisms of EDC-based crosslinking of antibodies under different conditions, which can substantially improve the analytical performance of immunodiagnostics and their cost-effectiveness.

## 2. Experimental Section

### 2.1. Materials

EDC, NHS, sulfoNHS and 2-(*N*-morpholino)ethane sulfonic acid (MES, pH 4.7), bovine serum albumin (BSA), 3,3',5,5'-tetramethylbenzidine (TMB) substrate kit, and bicinchoninic acid (BCA) protein assay kit were purchased from Thermo Fisher Scientific, USA. APTES, absolute ethanol, potassium hydroxide, 4-(2-hydroxyethyl)-1-piperazineethanesulfonic acid (HEPES), Tween 20, H_2_O_2_ (30%, v/v), Nunc 96-well flat bottom MTPs, H_2_SO_4_ (97.5%, v/v), horseradish peroxidase (HRP) and monoclonal anti-HRP antibody produced in mouse were procured form Sigma-Aldrich. The human Fetuin A/AHSG kit with all the necessary components was obtained from R&D Systems Inc., USA. All buffers, KOH and APTES solutions were prepared in 18 MΩ Milli-Q ultrapure water (UPW), while 0.1 M MES, pH 4.7 was employed to reconstitute EDC, NHS and sulfoNHS. It is to be noted that EDC, NHS and sulfoNHS were all freshly prepared for this study. The aqueous EDC, EDC/NHS and EDC/sulfoNHS mixtures are quite unstable and need to be used immediately or stored at −20 °C. 

Surface Plasmon Resonance was performed on BIAcore 3000 from GE Healthcare, Uppsala, Sweden. The surface interaction analysis (SIA) kit (BR-1004-05), containing SPR Au chips, was procured from GE Healthcare, U.K. The SPR Au chip was assembled according to the instructions supplied by the manufacturer. 10 mM HEPES-buffered saline (HBS) buffer, pH 7.4 was used as the running buffer for BIAcore and for making sample dilutions. The dilutions of HFA were made in BSA-preblocked glass vials, prepared by incubating with 1% (w/v) BSA for 30 min, to minimize analyte loss due to non-specific adsorption on sample tube surfaces and/or compromised immunogenicity [19]. The sandwich ELISA kits for human lipocalin-2 and human albumin were procured from R&D Systems, USA and Bethyl Labs, USA, respectively. The absorbance readings were taken by Tecan Infinite M200 Pro MTP reader. All the immunoassay procedures, employing the various EDC-based crosslinking chemistries on APTES-functionalized platforms, were performed at the same time under same ambient conditions and using same chemicals/consumables/instruments. Therefore, no experimental errors were induced due to experimental procedures and environment. Moreover, the analytical procedures employed for the various EDC-based crosslinking chemistries in this study have already been optimized [1].

### 2.2. Various EDC-Based Antibody Immobilization Strategies

Anti-HFA antibody (990 µL of 100 µg/mL in HBS) was incubated at RT for 15 min with 10 µL of the crosslinking solutions, *i.e.*, 10 µL of EDC (4 mg/mL); 5 µL of EDC (8 mg/mL) + 5 µL of NHS (22 mg/mL); and 5 µL of EDC (8 mg/mL) + 5 µL of sulfoNHS (22 mg/mL), for each of the three EDC-based crosslinking chemistries. This led to the activation of carboxyl groups on anti-HFA antibodies with EDC. It should be noted that the concentration of EDC employed was exactly the same, *i.e.*, 4 mg/mL in 10 µL of crosslinking solutions, in all three chemistries. Thereafter, the EDC-/EDC-NHS/EDC-sulfoNHS-activated anti-HFA antibodies were bound to the APTES-functionalized MTPs and SPR chips by following our previously developed incubation- and microfluidics-based immunoassay procedures for sandwich ELISA [1] and SPR-based immunoassays [13,14], respectively.

### 2.3. SPR-Based HFA Immunoassay

The SPR-based HFA immunoassay was performed using our previously developed immunoassay procedure on APTES-functionalized SPR Au chip [1], which involved sequentially the cleaning of SPR Au chip with freshly prepared piranha solution, APTES functionalization, immobilisation of anti-HFA antibody using various EDC-based crosslinking strategies, blocking with 1% BSA, and HFA detection (0.6–20 ng/mL^−1^). 

Initially, the SPR Au chip was cleaned by treating with freshly prepared piranha solution (60 µL of H_2_SO_4_ (97.5%, v/v): 30 µL of H_2_O_2_ (30%, v/v)) for 2 min followed by extensive rinsing with UPW. (Note: this was done outside the BIAcore instrument under the fume cabinet). The cleaned chip was then functionalized with APTES by incubating with 100 µL of 2% (v/v) APTES for 1 h at room temperature (RT) under the fume cabinet followed by extensive washing with UPW. 

The EDC-/EDC-NHS/EDC-sulfoNHS-activated anti-HFA antibodies were prepared by the procedure mentioned in Section 2.2. The flow rate used for all the process steps in the BIAcore 3000 was 10 µL/min. The antibody immobilization was done by injecting 50 µL of EDC-/EDC-NHS/EDC-sulfoNHS-activated anti-HFA antibody over all the four flow cells of SPR chip. The anti-HFA antibody bound chip was then blocked by injecting 20 µL of 1% (w/v) BSA. Subsequently, 50 µL of the dilution buffer *i.e.*, 10 mM HBS, pH 7.4 was passed through all the flow cells and the changes in SPR response units (RU) of all the four flow cells were determined. Finally, 50 µL of HFA at six different dilutions, *i.e.*, 0.6, 1.2, 2.5, 5, 10 and 20 ng/mL, were passed through the flow cells. The RU values of the blanks were then subtracted from the RU values of HFA detected in the corresponding flow cells. 

All the experiments were repeated four times in different flow cells. The error bars represent standard deviation. The HFA detection curves were plotted by SigmaPlot software (version 11.2) using four-parameter logistic function based on the standard curve analysis. 

### 2.4. HFA Sandwich ELISA

The HFA sandwich ELISA was similarly performed using our previous procedure [13,14], which involved sequentially the APTES-functionalization of MTPs, immobilisation of anti-HFA antibody using various EDC-based crosslinking strategies, blocking with 1% BSA, HFA detection (4.8 pg/mL^−1^–20 ng/mL^−1^), binding of biotinylated anti-HFA detection antibody to bound HFA, binding of HRP-conjugated streptavidin to biotinylated anti-HFA detection antibody, TMB substrate assay, and measuring the absorbance by Infinite M200 Pro microplate reader (Tecan, Austria) at a primary wavelength of 450 nm with a reference wavelength of 540 nm. All the experiments were done in triplicate. The error bars represent standard deviation. The absorbance of the control, *i.e.*, 0 ng/mL^−1^, was subtracted from all the assay values. The results were plotted by Sigma Plot software (version 11.2) using four-parameter logistic function based on the standard curve analysis. The sandwich ELISAs for human Lipocalin-2 and human albumin, and the direct ELISA for HRP were also done by the same procedure. 

## 3. Results and Discussion

**Figure 1:**
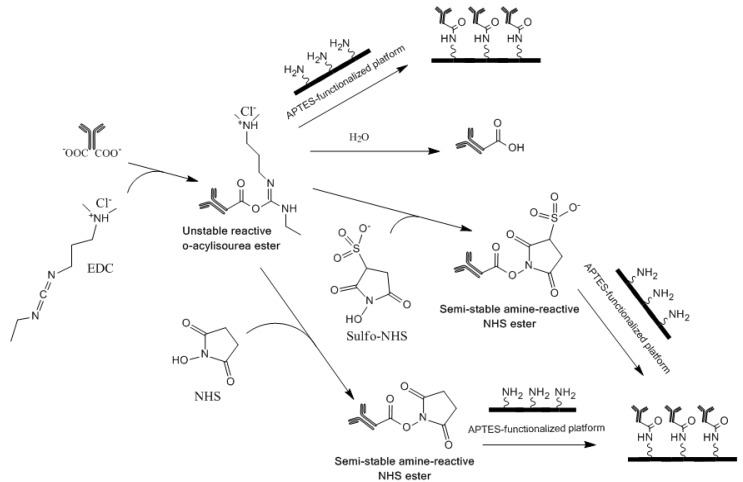
Schematic for the various 1-Ethyl-3-(3-dimethylaminopropyl) carbodiimide (EDC)-based chemistries that were employed to crosslink antibodies to antibodies on 3-aminopropyltriethoxysilane (APTES)-functionalized platforms for immunodiagnostic applications.

EDC is a carboxyl and amine-reactive zero-length crosslinker, which reacts initially with the carboxyl group and forms an *O*-acylisourea intermediate [5,20]. This intermediate reacts quickly with an amino group to form an amide bond and releases an isourea by-product (Figure 1). The *O*-acylisourea intermediate is unstable in aqueous solutions and gets hydrolyzed if it fails to react with an amine. The hydrolysis of the intermediate regenerates the carboxyl group and releases *N*-substituted urea. Therefore, NHS or sulfoNHS is required for stabilization as they react with the unstable reactive *O-*acylisourea ester intermediate to form semi-stable amine-reactive NHS ester that is stable for few hours at pH 7.4 [20] (Figure 1). The best crosslinking results are obtained when NHS-activated antibodies are used promptly for reacting to amine-functionalized substrates. The product information brochure for NHS and sulfoNHS [21] states that the activation of EDC-bound antibody with NHS decreases its water-solubility, while its activation with sulfoNHS preserves/increases its water-solubility. It further states that although NHS and sulfoNHS are not required for EDC-based heterobifunctional crosslinking of carboxyl and amino groups, their use enhances the coupling efficiency. The reaction of NHS- or sulfoNHS-activated antibodies with amines is also stated to be the most efficient at pH 7–8 [20,21]. 

EDC, NHS and sulfoNHS were prepared freshly in MES buffer, pH 4.7, which is a non-amine and non-carboxylate buffer, and thus, cannot interfere with the crosslinking of antibodies. However, in the present study, we employed 10 µL of EDC, EDC/NHS or EDC/sulfoNHS (prepared in 0.1 M MES, pH 4.7) and mixed it with 990 µL of antibody (prepared in 0.1 M PBS, pH 7.4). So the final pH of the crosslinking solution was close to the normal pH of 7.4, which is the most desirable pH for immunoassays. The crosslinking solution was incubated for 15 min at RT, which resulted in the binding of EDC to the carboxyl group of anti-HFA antibody followed by the subsequent binding of EDC-activated anti-HFA antibody to NHS or sulfoNHS to form EDC/NHS or EDC/sulfoNHS activated anti-HFA antibody. The EDC or EDC/NHS or EDC/sulfoNHS activated anti-HFA antibodies were then provided to APTES-functionalized bioanalytical platforms, which led to the crosslinking of anti-HFA antibodies to the free amino groups on the surface. 

**Figure 2:**
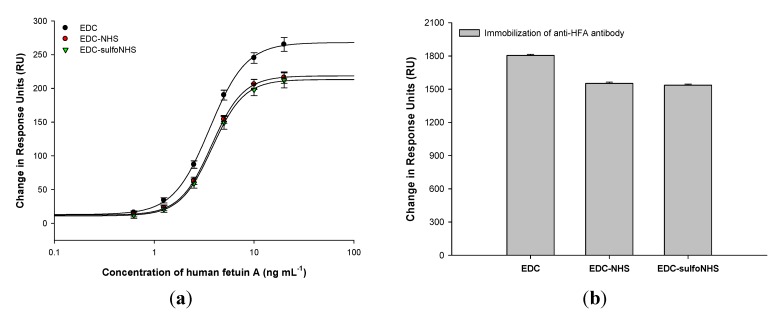
(**a**) Surface plasmon resonance (SPR)-based anti-human fetuin A (HFA) immunoassay using various EDC-based strategies for crosslinking anti-HFA antibodies to APTES-functionalized SPR gold chips; (**b**) SPR response units corresponding to the binding of capture anti-HFA antibodies by the various strategies. The values are average of four repeats in different flow cells. The errors bars represent standard deviation.

**Figure 3:**
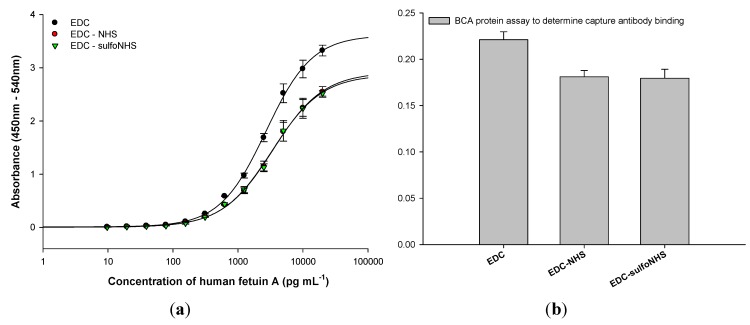
(**a**) HFA sandwich sandwich enzyme linked immunosorbent immunoassay (ELISA) using various EDC-based strategies for crosslinking anti-HFA antibodies to APTES-functionalized 96-well microtiter plates (MTP); (**b**) bicinchoninic acid (BCA) protein assay to determine the amount of anti-HFA antibody bound to MTP by various strategies. All experiments were done in triplicate. The errors bars represent standard deviation.

**Figure 4:**
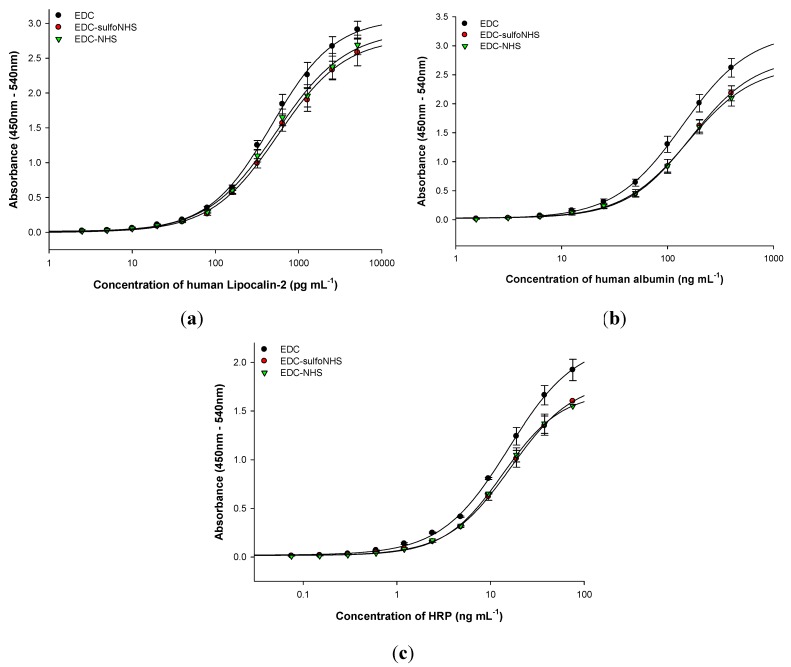
(**a**) Human Lipocalin-2 sandwich ELISA; (**b**) human albumin sandwich ELISA; and (**c**) horseradish peroxidase (HRP) direct ELISA, using various EDC-based strategies for crosslinking capture antibodies to APTES-functionalized MTPs. All experiments were done in triplicate. The errors bars represent standard deviation.

The SPR-based HFA immunoassays (Figure 2(a)) performed on APTES-functionalized SPR Au chip using the various EDC-based antibody crosslinking strategies further demonstrated that the anti-HFA antibody immobilization density using EDC was 17 ± 0.1% higher than that using EDC/NHS or EDC/sulfoNHS (Figure 2(b)). The higher antibody immobilization density was responsible for the enhanced HFA detection in case of EDC. Similar results were obtained in the HFA sandwich ELISA on APTES-functionalized MTPs (Figure 3). The amount of antibody bound to MTP, determined by BCA protein assay, also provided similar results of 22 ± 0.8% higher antibody binding in case of EDC. Therefore, the anti-HFA immobilization density by EDC was on an average 19.5% higher than that of EDC-NHS or EDC-sulfoNHS (taking the mean of SPR-based HFA immunoassay and BCA protein assay). Similar experiments were performed for sandwich and direct ELISAs for other analytes. The human lipocalin-2 and human albumin were detected by sandwich ELISA, while HRP was detected by direct ELISA (Figure 4). The EDC-based antibody crosslinking was observed to be the best in all immunoassays on APTES-functionalized platforms in terms of analyte detection, thereby demonstrating better analytical performance. But the limit of detection, linearity, dynamic range and half-maximal effective concentration of the assay curves were not much affected. Thus, these results will enhance the cost-effectiveness of immunoassays without sacrificing the analytical performance. 

The present study demonstrates that the EDC-based crosslinking of antibodies is unaffected by the instability of reactive *O-*acylisourea ester intermediate, if EDC-activated antibodies are immediately allowed to react with the free amino groups on APTES-functionalized platforms. However, EDC/NHS and EDC/sulfoNHS were found to have lower coupling efficiencies than EDC, which is contrary to the product guidelines and literature stating the enhanced coupling efficiencies when EDC is used along with NHS or sulfoNHS [20,21]. As the molecular mechanisms responsible for this behavior are unknown, we expect that this may be due to the molecular interactions between EDC and NHS/sulfoNHS, which might be interfering with the binding of EDC-activated antibodies to the free amino groups on APTES-functionalized bioanalytical platforms. The semi-stable amine-reactive NHS ester formed by EDC-NHS/EDC-sulfoNHS may also be less efficient than the unstable reactive *O-*acylisourea ester formed by EDC in binding to the amino groups. Moreover, the decreased antibody crosslinking by EDC-NHS or EDC-sulfoNHS in comparison to EDC might also be due to the cross-reaction between activated antibodies, which is highly favorable as it occurs in the homogenous phase. On the other hand, the reaction of the activated antibody with the amino groups on APTES-functionalized surface occurs in the heterogeneous phase with limited mass transfer. As the NHS ester is more reactive than the EDC ester, it could lead to the formation of antibody aggregates, thereby preventing their binding to the APTES-functionalized surface. The possibility of these polymerisation reactions was mentioned by Wong *et al*., 2009 [3]. Therefore, more intensive research efforts are required to determine the exact cause of the observed results.

The crosslinking of antibody in a particular EDC-based crosslinking strategy is dependent on the molar ratios of EDC, NHS or sulfoNHS and antibody; the buffer type/concentration; pH; and, the isoelectric point of antibody [6,22]. EDC gives optimal coupling at pH 4.5–5.0 *i.e.*, in the acidic conditions, which is typically lower than the normal pH of 7–8 that one wish to conjugate. The mechanism of EDC-based crosslinking of antibodies was proposed by Nakajima and Ikada [23]. The NHS esters produced by EDC/NHS and EDC/sulfoNHS were proposed to be more stable than the *O*-acylisourea ester produced by EDC. The EDC/NHS or EDC/sulfoNHS based crosslinking was also specified to be optimal at pH 7.2–8.5. The isoelectric points (pI) of most IgGs are in the range of 7.4–8.6 [24]. Therefore, the employed pH of about 7.4 in the present study for various EDC-based strategies is within the desired range of 0.5–1.0 pH unit below the pI of IgGs. 

Our results are contrary to the previous reports [25] that state lesser binding efficiency for EDC at a normal pH of 7.4 in comparison to that of EDC/NHS or EDC/sulfoNHS. Recently, the EDC-based crosslinking of antibody to carboxylated quantum dots has been demonstrated to be optimal at pH 10.8 and a new mechanism for EDC-based amine-carboxyl coupling under basic aqueous conditions was proposed [22]. Therefore, our present study strongly suggests the need for intensive research efforts to unravel the exact mechanisms that are responsible for the EDC-based crosslinking under different experimental conditions. It will be immensely useful to researchers in improving the analytical performance of immunodiagnostics and other bioanalytical applications, where the EDC-based crosslinking chemistries and APTES-functionalized platforms are being extensively used. However, the antibody crosslinking is highly dependent on the particular EDC/protein couple. Therefore, the results of this study on selected set of antibodies cannot be generalized and transposed to all EDC/protein couples.

## 4. Conclusions

EDC, EDC-NHS and EDC-sulfoNHS were employed to crosslink anti-HFA antibodies on APTES-functionalized platforms for immunodiagnostic applications. The SPR immunoassay and sandwich ELISA for HFA demonstrated more efficient antibody crosslinking by EDC in comparison to EDC-NHS and EDC-sulfoNHS at a normal pH of about 7.4, which increased the analytical performance and cost-effectiveness of our immunoassays. Similar results were achieved in sandwich ELISAs for human Lipocalin-2 and human albumin, and direct ELISA for HRP. We believe that the present study will be indispensable for triggering the quest in researchers for an in-depth investigation into the mechanisms of EDC-based amine-carboxyl coupling under different experimental conditions, which will provide a guided insight into the EDC-based coupling chemistries for the development of improved immunodiagnostics. 
